# Screening a library of temperature-sensitive mutants to identify secretion factors in *Staphylococcus aureus*

**DOI:** 10.1128/jb.00433-24

**Published:** 2025-01-16

**Authors:** Owen Leddy, Amany M. Ibrahim, Muhammad S. Azam, Sadie Solomon, Wenqi Yu, Olaf Schneewind, Dominique Missiakas

**Affiliations:** 1 Department of Microbiology, Howard Taylor Ricketts Laboratory, The University of Chicago 456539 , Chicago, Illinois, USA; University of Notre Dame, Notre Dame, Indiana, USA

**Keywords:** *Staphylococcus aureus*, temperature-sensitive mutant, protein secretion, SecA, SecG, PepV

## Abstract

Protein secretion is an essential cell process in bacteria, required for cell envelope biogenesis, export of virulence factors, and acquisition of nutrients, among other important functions. In the Sec secretion pathway, signal peptide-bearing precursors are recognized by the SecA ATPase and pushed across the membrane through a translocon channel made of the proteins SecY, SecE, and SecG. The Sec pathway has been extensively studied in the model organism *Escherichia coli*, but the Sec pathways of other bacteria such as the human pathogen *Staphylococcus aureus* differ in important ways from this model. Unlike in *E. coli*, a subset of precursors in *S. aureus* contains a YSIRK/GXXS (YSIRK) motif in an extended signal peptide. These proteins are secreted into the cross-wall compartment bounded by invaginating septal membranes during cell division. To gain insights into the factor(s) and mechanism(s) enabling protein secretion and spatial specificity in *S. aureus*, we isolated and screened a collection of temperature-sensitive (*ts*) mutants. These efforts identified at least one *secA(ts*) allele as well as mutations in the *secG* and *pepV* genes. A SecA pull-down experiment identified SecDF, all ribosomal proteins, several chaperones and proteases, as well as PepV, validating the genetic screen in identifying candidate cofactors of SecA in *S. aureus*.

**IMPORTANCE:**

All organisms use the Sec pathway for protein secretion, and key components of this pathway are essential for viability. The discovery of conditional loss-of-function mutants played an important role in defining the genetic basis of protein secretion in model organisms. In turn, the identification of Sec components facilitated mechanistic studies and revealed general rules for protein secretion but did not answer species-specific intricacies. Gram-positive bacteria, such as *Staphylococcus aureus*, restrict the secretion of some proteins into the septal membranes that bind their division site at mid-cell. Here, we screen a library of conditional temperature-sensitive mutants to define components of the Sec pathway of *S. aureus* and factors that may regulate its activity.

## INTRODUCTION

Earlier studies to decipher the mechanism of protein translocation into and across membranes identified shared and conserved features among secreted proteins and among organisms. Both in bacteria and eukaryotes, signal sequences or stop transfer sequences were found to direct protein precursors to the bacterial plasma membrane or the rough endoplasmic reticular membrane of eukaryotes, in a process involving energy (1–5). The notion that specific topogenic sequences could be used to direct proteins out of the cytosol was first formulated as the signal hypothesis before the machinery for secretion was known (5). The identification of cleavable amino-terminal signal peptides prompted the search for conserved elements within signal sequences (*cis* elements) and machineries (*trans* elements) for the recognition, export, and processing of presecretory proteins (6–8). Both biochemical and genetic approaches were used to identify *cis* and *trans* elements governing protein secretion (8, 9).

In *Escherichia coli*, genetic approaches involved the development and use of translational hybrids between *lacZ* and genes of secreted components such as the maltose transporter, periplasmic maltose-binding protein (MBP), outer membrane protein porin (LamB, λ receptor), and the inner membrane protein, MalF (10, 11); transport of LacZ hybrids would result in the loss of β-galactosidase activity unless *cis* and *trans* elements were to be genetically altered (12, 13). Unexpectedly, some of these hybrids interacted with the transport system in an irreversible manner resulting in loss of viability (12, 13). Suppressors that restored bacterial growth were mapped to the signal sequence, altering the hydrophobicity or charge of the segment and, thus, corroborating the signal hypothesis of Blobel and Dobberstein (5). Toxicity could also be reverted through the acquisition of extragenic suppressor mutations that were first mapped to the *secA* gene (14–16). For example, the *E. coli* suppressor strain MM52 displays a temperature-sensitive (*ts*) phenotype and carries a conditional lethal mutant in the *secA* gene that accumulates precursors of secreted proteins at the nonpermissive temperatures (14, 17). Additional *ts* alleles with similar secretion defects were mapped to *secB* and *secY* (18, 19). Signal sequence mutants were exploited to identify extragenic suppressors that could restore export of LamB to its normal outer membrane location and mapped to SecY (PrlA) (20).

Such genetic approaches have not systematically been performed for other microbes including *Staphylococcus aureus*. Rather, comparison of sequences has been used to identify homologous Sec factors. *S. aureus* is a Gram-positive coccus that contains several *sec* genes and evolved both shorter and longer signal peptides (*cis* elements). Some of the longer signal peptides are endowed with the so-called YSIRK/GXXS (YSIRK) motif that is necessary and sufficient for the secretion of precursors into septal membranes. *Trans* elements restricting such spatial secretion remain to be identified. Here, chemical mutagenesis was used to isolate and screen mutants with a temperature-sensitive (*ts*) growth phenotype since protein secretion is also essential for *S. aureus* viability (21). *ts* mutants were subsequently analyzed for defects in the secretion of proteins with and without a YSIRK motif. Whole-genome sequencing and complementation studies were performed to identify and characterize mutations associated with secretion defects. Affinity chromatography was also used to identify SecA binding partners that might be involved in septal targeting, preprocessing, or secretion of precursors containing a YSIRK motif.

## RESULTS

### Isolation and screening of temperature-sensitive mutants for defect in protein secretion

**Fig 1:**
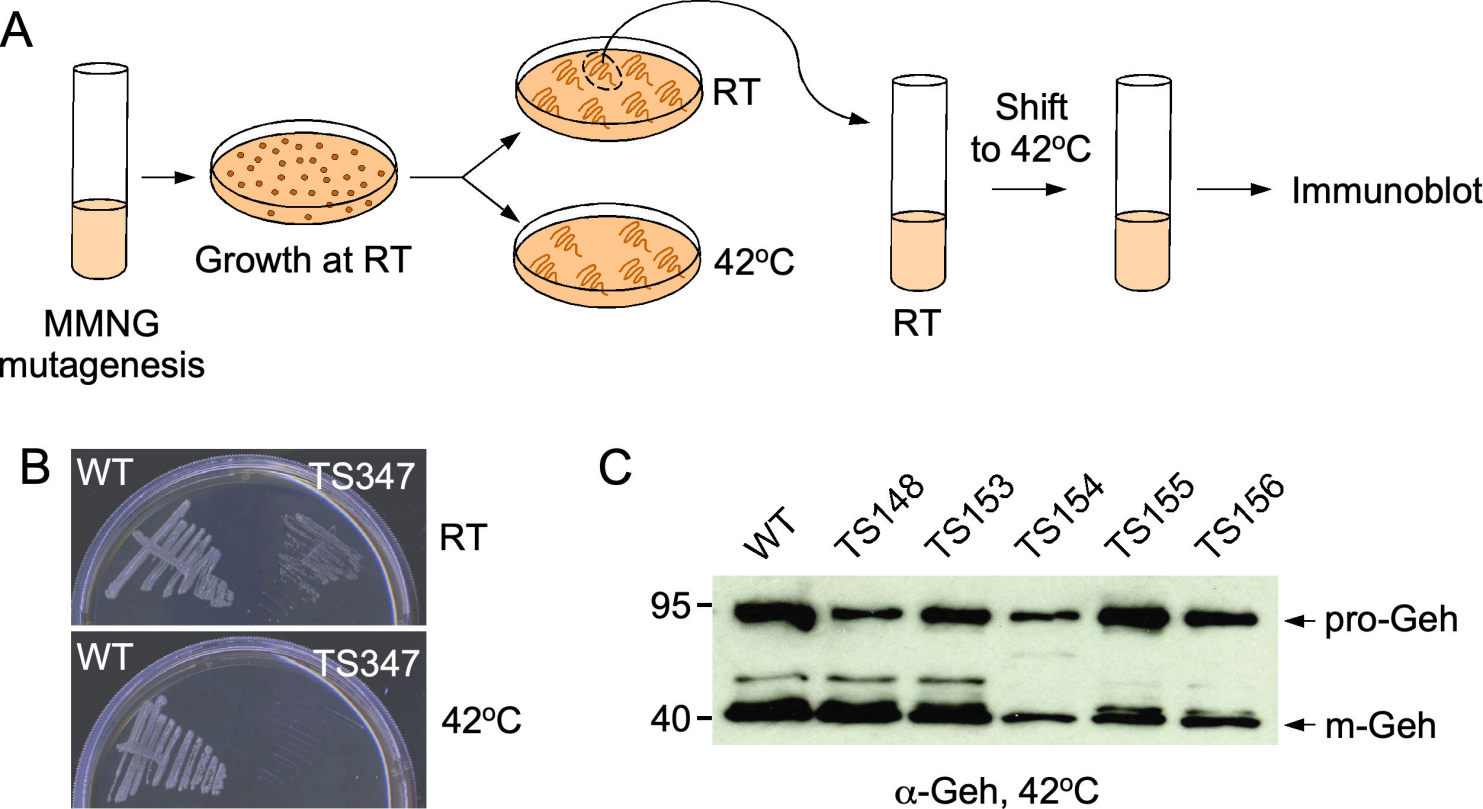
A genetic screen for temperature-sensitive mutants with defect for protein secretion in *Staphylococcus aureus*. (**A**) Schematic representation of a method for isolating temperature-sensitive mutants and screening for protein secretion defects. (**B**) TS 347 isolate grown on agar at room temperature (RT; top) and 42°C (bottom) compared to wild type (WT) RN4220. (**C**) Example of an immunoblot examining the presence of Geh in the supernatants of cultures grown at 42°C. Arrows indicate the positions of pro- (72 kDa) and mature (42 kDa) mGeh.

To generate a library of mutants and screen for conditional defects in growth and protein secretion, methylnitronitronitrosoguanidine (MNNG) was used to mutate the wild-type *S. aureus* strain RN4220 (Fig. 1A). Mutagenized bacterial populations were grown at room temperature (20°C), and approximately 30,000 colonies were streaked in duplicate on two plates that were subsequently incubated at room temperature and 42°C, respectively (Fig. 1A and B). Two hundred and ten *ts* mutants that failed to grow at 42°C were isolated.

**Fig 2:**
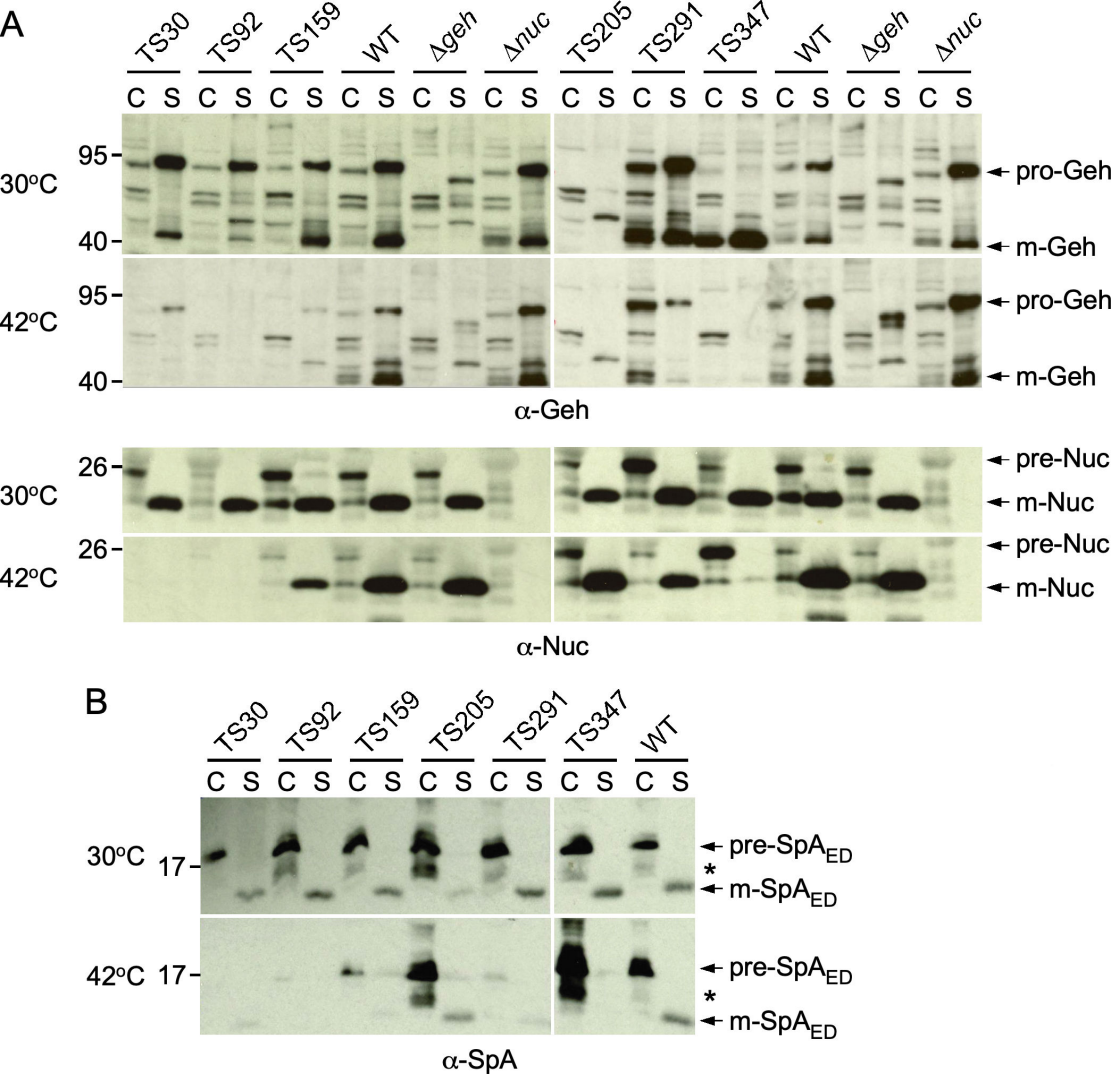
Analysis of secretion profiles of six TS isolates. Bacterial cultures of TS isolates 30, 92, 159, 205, 291, and 347 grown at 30°C or 42°C were separated to obtain cellular (C) and secreted (S) fractions. Proteins in these fractions were separated by SDS-PAGE and transferred to membranes for immunoblotting with polyclonal sera raised against Geh and Nuc (A; αGeh, αNuc) or SpA (B; αSpA). TS isolates in panel B carried a plasmid expressing a truncated SpA variant (SpA_ED_) that is released in the culture medium following secretion. Arrows indicate the positions of pro-Geh, precursors (pre) of Nuc and SpA_ED_, and fully processed, mature (m) species. Molecular weight of these various species are as follows: pro-Geh, 72 kDa; m-Geh, 42 kDa; pre-Nuc, 25 kDa; m-Nuc, 19 kDa; pre-Spa_ED_, 17 kDa; and m-Spa_ED_, 13 kDa, respectively. An asterisk also indicates an intermediate migrating SpA_ED_ species observed in C fractions; the nature of this species is not known.

To assess whether any of the mutants with a *ts* growth phenotype were also conditionally impaired in protein secretion, each isolate was grown overnight at permissive temperature. Cells were sedimented and resuspended in prewarmed medium for incubation at permissive and nonpermissive temperatures for 3 hours. Cultures were normalized to the same optical density and spun down to separate cells (C) and secreted proteins (S). Proteins in the S fractions were separated by SDS-PAGE and transferred to membranes for immunoblotting of the secreted YSIRK protein glycerol ester hydrolase (Geh). A representative secretion analysis for some of these mutants is shown in Fig. 1C. Six candidates, TS30, TS92, TS159, TS205, TS291, and TS347, were further analyzed to compare secretion of nuclease (Nuc) and Geh (Fig. 2A). Unlike Geh, the Nuc precursor lacks the YSIRK motif and served to distinguish mutants with general secretion defects from those with YSIRK-specific defects (22–25). Of note, Geh is produced as a pre-pro-enzyme (pre-pro-Geh), but this species is not detected with our polyclonal serum. Secreted Geh is detected as both pro-Geh and mature Geh (mGeh). To probe further for defects in secretion of proteins containing YSIRK motifs, the six *ts* isolates were transformed with a plasmid expressing a truncated SpA variant composed only of the N-terminal signal sequence, which contains a YSIRK motif, and the first two IgG-binding domains, E and D (SpA_ED_) (Fig. 2B). Full-length SpA is otherwise tethered to peptidoglycan by the sortase A enzyme (24, 25). Briefly, in addition to being severely impaired for growth at 42°C, isolates TS30, TS92, and TS159 produced greatly reduced amounts of Geh, Nuc, and SpA_ED_ preempting any straightforward conclusion for protein secretion (Fig. 2A and B). TS205 exhibited a conspicuous lack of Geh but did not display any defect in the secretion of Nuc (Fig. 2A). However, the immune-reactive species migrating between pre- and mature SpA_ED_ and marked with an asterisk appeared to accumulate in the cytoplasm of TS205 (Fig. 2B); we do not know what this species might be, but it could represent an intermediate processing product of YSIRK signal sequences. Both Nuc and SpA_ED_ accumulated in the cytoplasm of TS347 at 42°C, as visualized by immune-reactive species of slightly greater sizes and labeled pre-Nuc and pre-SpA_ED_ (Fig. 2A and B). The amount of SpA_ED_ noted with an asterisk also increased in the sedimented C fraction of TS347 at 42°C (Fig. 2B). Together, these results suggest that TS347 exhibits a general secretion defect at nonpermissive temperature. TS291 displayed a selective defect for secretion of the two YSIRK substrates, Geh and SpA_ED_, but continued to secrete Nuc (non-YSIRK) at the nonpermissive temperature (Fig. 2A and B). At permissive temperature, both pre-Geh and mature Geh are observed in the sedimented fraction (C) of TS291 suggesting that these species may remain associated with these cells; similarly, mature Geh appears to sediment with cells of TS347 (Fig. 2A). Together, these observations validate that our *ts* mutant screening approach can isolate mutations that affect secretion of YSIRK motif-containing proteins specifically and mutations that impair *S. aureus* protein secretion generally.

### Whole-genome analysis of TS isolates

To identify mutations that may be responsible for the phenotype of *ts* isolates with defects in protein secretion, chromosomal material was extracted from the mutants and parent strain RN4220 and subjected to sequencing. The nucleotide sequences of *Staphylococcus aureus* NCTC 8325, accession number CP000253 in the PubMed Databank, were used as the reference sequence to identify genes with single nucleotide polymorphisms (SNPs) in each TS isolate (Table S1). This analysis revealed that each isolate carried multiple SNPs, with at least one per isolate in an essential gene, possibly accounting for the observed conditional growth phenotypes. Isolates TS92 and TS159 carried a large number of SNPs and were eliminated from further investigations.

Of the remaining isolates, TS291 had the largest number of SNPs, with eight found in genes essential for growth (Table S1). We hypothesized that a mutation in *ltaA*, resulting in amino acid substitution proline 201 to serine (P201S), could possibly account for the reduced secretion of YSIRK motif-containing proteins (Fig. 2A and B; Table S1) (26). *ltaA* encodes a diglucosyl-diacylglycerol (Glc_2_-DAG) flippase involved in the synthesis of lipoteichoic acid (LTA), and *ltaA* mutants fail to restrict the secretion of YSIRK motif-containing precursors at the septum (26).

TS30, TS205, and TS347 carried 8, 9, and 9 SNPs, respectively. SNPs in essential genes included those encoding DNA primase (TS30), GTPase ObgE and D-alanyl-alanine synthetase A (TS205), and SecA, GidA, and TrmE (TS347) (Table S1). TS205 also carried SNPs resulting in the introduction of stop codons at amino acids Q40 of *secG* and Q110 of *geh*, the latter explaining the absence of Geh production observed for this isolate (Fig. 2A). Strain TS30 did not display a stable *ts* phenotype upon recovery from frozen stocks, preventing further studies with the isolate after whole-genome sequencing. This isolate also bore mutations in genes SAOUHSC_01868 and SAOUHSC_02008, which were further investigated for their possible contribution to protein secretion in *S. aureus*. TS347, which displayed a general secretion defect, carried two notable SNPs resulting in amino acid substitutions G187D in SecA, SecA_G187D_, and G29E in SecG, SecG_G29E_. SecG is a component (along with SecY and SecE) of the pore that mediates the translocation of SecA-bound substrates across the plasma membrane. We therefore hypothesized that amino acid substitutions in SecG and/or SecA were responsible for the *ts* growth and protein secretion phenotypes of TS347.

### The thermosensitive phenotype and secretion defect of TS347 are caused by the *secA_G187D_* allele

**Fig 3:**
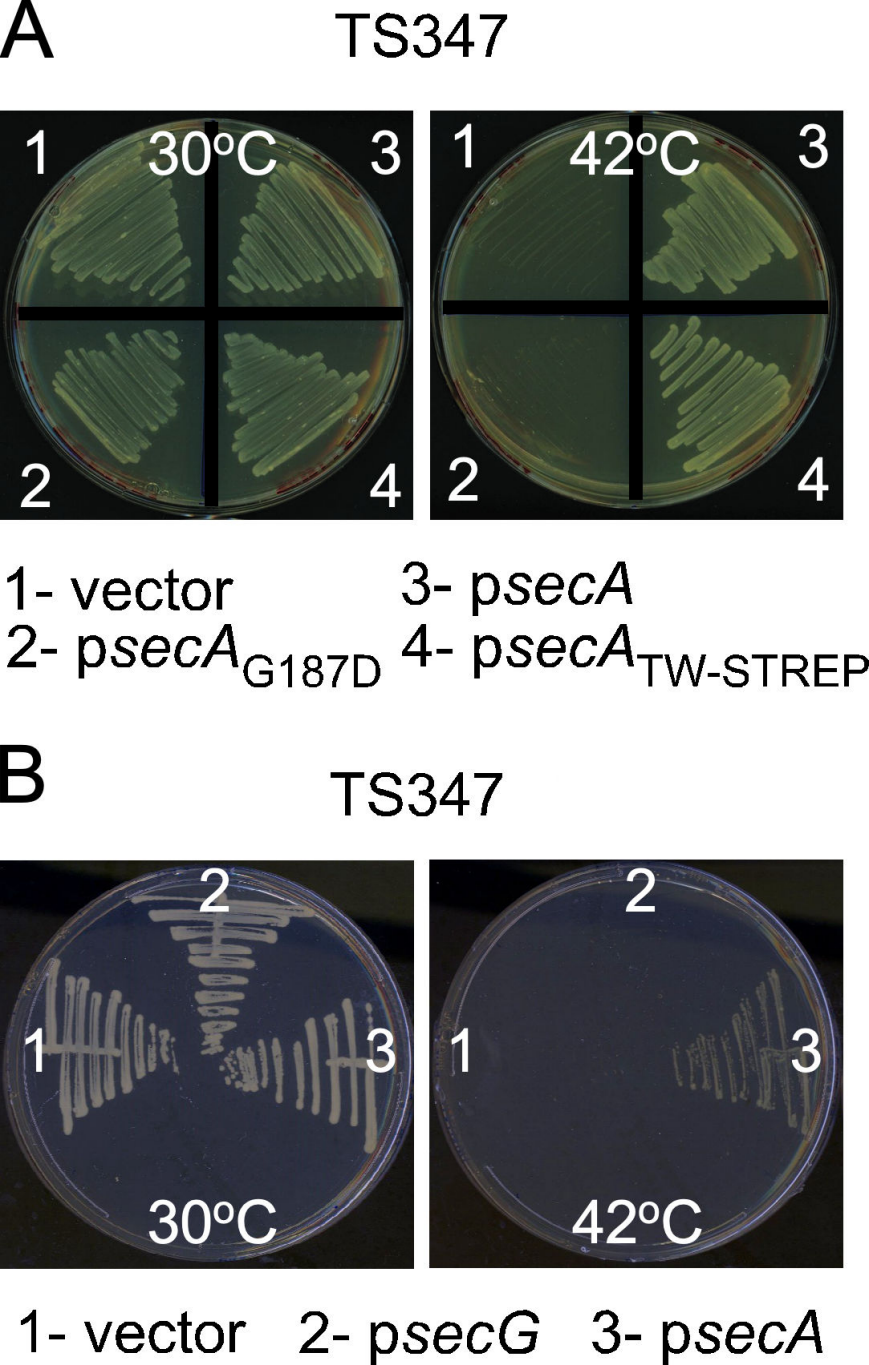
Genetic complementation of TS347. (**A and B**) Bacterial strains TS347 carrying empty vector, plasmid-encoded SecA (p*secA*; WT *secA*), SecA_G187D_ (p*secA*_G187D_; G187D *secA* variant), and SecA_TW-STREP_ (p*secA*_TW-STREP_; WT *secA* with a C-terminal twin [TW]-STREP tag sequence) (**A**) or SecG (p*secG*; WT *secG*) (**B**) were plated at permissive (30°C) and nonpermissive temperature (42°C) to assess the bacterial growth.

**Fig 4:**
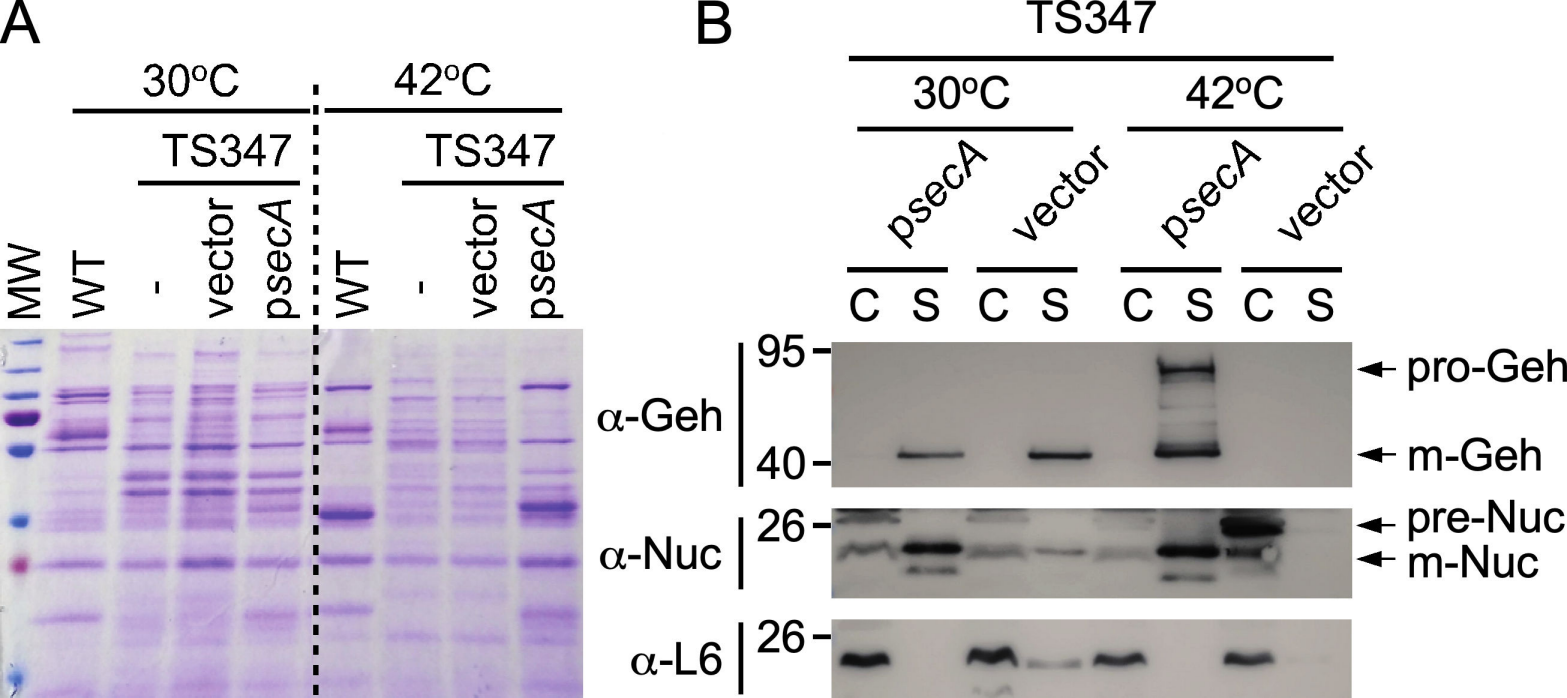
Phenotypic complementation of TS347 with plasmid-encoded SecA. Cultures grown at 30°C and 42°C were fractionated into cells (C) and secreted proteins (S) and separated by SDS-PAGE. (**A**) Proteins in S fractions of 30°C/42°C cultures from wild-type (WT, RN4220) or TS347 carrying nothing (-), vector, or p*secA* were visualized by Coomassie staining. (**B**) Proteins in C and S fractions of 30°C/42°C cultures from TS347 carrying vector or p*secA* were transferred to membranes for immunoblot analyses with αGeh, αNuc, or αL6 (ribosomal protein L6) polyclonal sera. L6 served as fractionation control.

**Fig 5:**
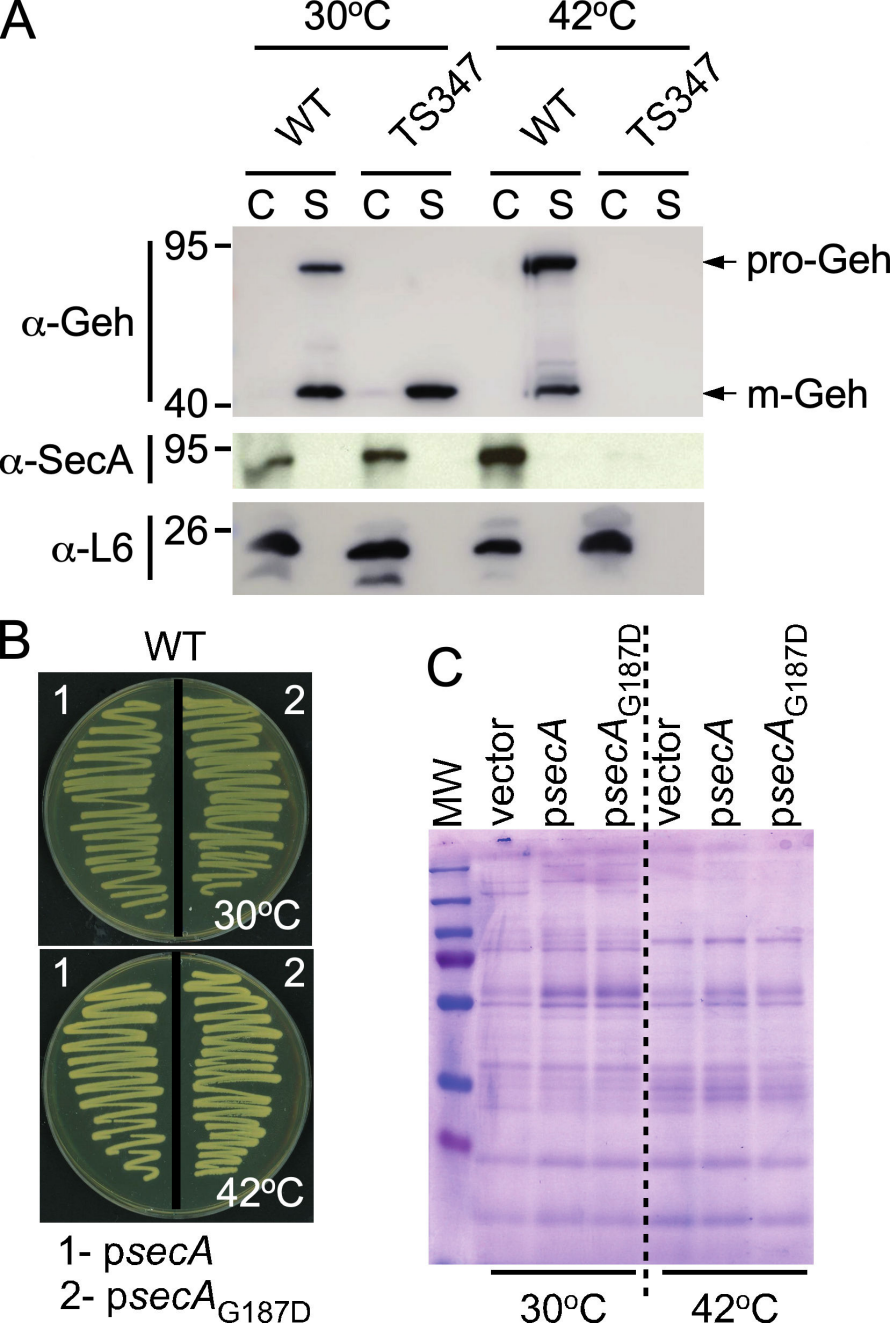
SecA_G187D_ is unstable at nonpermissive temperature and does not exert a dominant negative phenotype. (**A**) Immunoblot identifying SecA in bacterial extracts. Cultures grown at 30°C and 42°C were fractionated into cells (C) and secreted proteins (S) and fractions examined by immunoblot for production of SecA, L6, and secreted Geh using cognate polyclonal sera. L6 served as fractionation control. (**B**) Wild-type (WT) RN4220 strains carrying plasmid-encoded SecA (p*secA*) or SecA_G187D_ (p*secA*_G187D_) were plated at permissive (30°C) and nonpermissive temperature (42°C). (**C**) Proteins in S fractions of 30°C/42°C cultures from wild-type (WT, RN4220) carrying vector, p*secA* or p*secA*_G187D_, were visualized by Coomassie staining.

Showing that *ts* mutations in known protein secretion pathway components are responsible for the phenotype of TS347 would validate our screening approach. To determine whether SNPs in SecA and/or SecG were responsible for the temperature-dependent growth and secretion defects observed in TS347, the isolate was subjected to a complementation analysis. Plasmid expression of wild-type *secA* (p*secA*) but not *secA*_G187D_ or empty vector control restored the growth defect of TS347 at 42°C (Fig. 3A). Plasmid expression of wild-type *secG* (p*secG*) did not restore growth of TS347 at nonpermissive temperature (Fig. 3B). Next, cultures grown at 30°C and 42°C were separated into cells (C) and supernatants (S). Secreted proteins in the “S” fractions were separated by SDS-PAGE and stained with Coomassie (Fig. 4A). C and S fractions were also transferred to membranes for immunoblot analyses (Fig. 4B). Coomassie staining shows that expression of wild-type *secA* on a plasmid restored protein secretion to a level comparable to that of RN4220 (Fig. 4A). Similarly, immunoblots revealed the presence of both Geh and Nuc in the “S” fraction of spun cultures of TS347/p*secA* grown at 42°C (Fig. 4B). To further examine the impact of G187D on SecA, a western blot was performed using extracts prepared from RN4220 and TS347. This analysis failed to detect SecA_G187D_ in cultures grown at 42°C (Fig. 5A), suggesting that SecA_G187D_ is unstable at the nonpermissive temperature. Next, plasmid-encoded *secA*_G187D_ (p*secA*_G187D_) was transformed into wild-type RN4220. SecA forms a dimer to bind precursors in an ATP-dependent manner. Oligomerization between wild-type SecA and a mutant with altered function would result in a dominant-negative phenotype at permissive temperature. However, neither growth nor protein secretion was affected upon overexpression of *secA*_G187D_ in wild-type RN4220 (Fig. 4B and C), indicating no dominant negative phenotype. We conclude that the thermosensitive growth phenotype and secretion defect of TS347 are caused by degradation of SecA_G187D_ at higher temperature, but SecA_G187D_ is otherwise stable and functional at permissive temperature.

### Analysis of additional alleles found in *ts* isolates

We next evaluated other SNPs found in *ts* isolates for either growth or secretion defects. Like TS347, TS205 carried a SNP in the *secG* gene. This SNP, C to T substitution at nucleotide position 784,343 (Table S1), resulted in the introduction of a stop codon at position Q40. While prior reports have used deletion of *secG* to show that the gene is dispensable for protein secretion in several bacteria, including *S. aureus* (27–29), allelic replacement was undertaken to produce a variant of RN4220 with a similar stop codon, *secG*_Q40STOP_. This was performed to rule out the possibility that other SNPs found in TS205 may have arisen as a compensation mechanism, for instance, to prevent a negative dominant interaction between truncated SecG and the SecYE complex. However, when expressed in RN4220 alone, the *secG*_Q40STOP_ allele did not cause any growth (Fig. S1A) or secretion defects (not shown).

Isolate TS30 carried the smallest number of SNPs but none of these mutations mapped to factors known to be involved in protein secretion. As noted above, the *ts* phenotype of the isolate was eventually lost possibly due to truncation of MutS; loss of *mutS* would be expected to cause defects in DNA repair causing a higher mutation rate (30), potentially explaining the loss of this isolate. Nonetheless, two SNPs seemed noteworthy. One SNP was identified in SAOUHSC_01868, a gene encoding a predicted dipeptidase referred to as PepV that is further characterized in the companion study. The second SNP resulted in amino acid substitution S29G in the uncharacterized protein SAOUHSC_02008. Sequence comparison in the Cluster of Orthologous Gene database identifies a match with COG1682, annotated as an ABC-type polysaccharide/polyol phosphate export permease (31). COG1682 is loosely related to TagG, the membrane transporter of wall teichoic acid. This allele was reconstituted in wild-type RN4220, but the resulting strain did not display any growth, teichoic acid, or protein secretion defects (not shown). We conclude that the SAOUHSC_02008 is unlikely to play a role in protein secretion, while PepV (SAOUHSC_01868) remained a novel candidate for further investigation.

Isolate TS291 carried too many SNPs for systematic analysis, yet this isolate displayed a selective YSIRK secretion defect. One of the SNPs resulted in a G to A substitution at nucleotide position 926,315 leading to a change from P to S in amino acid number 201 (*ltaA*_P201S_). Our recent studies identified the LTA assembly pathway as a contributor of septal secretion; specifically, septal secretion of Staphylococcal protein A is no longer restricted to the cross-walls in a mutant lacking *ltaA* (26). To examine if allele *ltaA*_P201S_ is a loss-of-function allele, the corresponding gene was amplified from genomic DNA of isolate TS291. The new plasmid p*ltaA*_P201S_ was transformed in a strain lacking *ltaA* (*ltaA::erm*/p*ltaA*_P201S_) for phenotypic comparison with *ltaA::erm* carrying empty vector or p*ltaA* (Fig. S1B). In the absence of *ltaA*, the cell polymerizes glycerol-phosphate (Gro-P)—the repeating unit of the LTA polymer, poly(Gro-P)—mostly onto diacylglycerol instead of diglucosyl-diacylglycerol. As a result, chains of poly(Gro-P) are longer in bacteria lacking *ltaA* as visualized by western blotting using a monoclonal antibody against LTA (32, 33). An analysis of LTA length by immunoblot demonstrated that p*ltaA*_P201S_ restored the wild-type length of LTA (Fig. S1). Thus, we conclude that the YSIRK secretion defect observed in strain TS291 cannot be attributed to an inactive *ltaA* allele.

### A pull-down assay to identify cofactors of SecA in *S. aureus*

SecA is required for the secretion of preproteins with both canonical and YSIRK-containing signal sequences, yet YSIRK precursors are secreted selectively into bacterial cross-walls, while other SecA substrates are not. It is possible that spatially restricted secretion in round cocci is governed through interactions between SecA and previously unidentified secretion factors. To identify novel binding partners that might regulate secretion, we generated a SecA variant with N-terminally fused twin strep tag sequence (TW-STREP tag) for affinity purification and transformed the corresponding plasmid, p*secA*_TW-STREP_, into TS347. Production of SecA_TW-STREP_ restored both growth (Fig. 3A) and secretion of Geh and Nuc at nonpermissive temperature (not shown). This experiment indicated that the tag does not alter the activity of SecA_TW-STREP_. Both the vector control and p*secA* (SecA without a tag) were transformed into RN4220 to perform a pull-down experiment using cleared cell lysates passed over commercially obtained Strep-Tactin Sepharose beads. Bound proteins were eluted with desthiobiotin and analyzed by liquid chromatography with tandem mass spectrometry (LC/MS/MS; Table 1; Table S2). As expected, this analysis identified SecA, as well as two cell division proteins, FtsZ and EzrA, that localize to the septum (Table 1). Several proteins involved in transcription and translation were also identified; and although not listed in the table, the 54 core bacterial ribosomal proteins, i.e., all 33 from the 50S subunit (L1 to L7/L12, L9 to L11, L13 to L25, and L27 to L36) and all 21 (S1 to S21) from the 30S subunit, were also identified (34). SecDF, trigger factor, and several chaperones, DnaK, GroEL, and subunits of Clp proteases were also highly represented (Table 1). Lastly, the mass spectrometry analysis identified SAOUHSC 1868, annotated as dipeptidase PepV. Given that the conditionally secretion-defective isolate TS30 bore a mutation in the gene encoding PepV, this convergence of biochemical and genetic evidence identified PepV as a potential hit from our screen and a candidate for further investigation to determine its contribution to protein secretion in *S. aureus*.

**TABLE 1:** List of non-ribosomal proteins identified by LC/MS/MS analysis of the SecA_TW-STREP_ purification experiment^*a*^

Reference identification	Gene symbol	Annotation
Secretory pathway, chaperone, proteases		
O06446_SECA1_STAA8	secA1	Protein translocase subunit SecA 1
Q2FXT8_Q2FXT8_STAA8	secDF	Protein-export membrane protein SecDF
Q2FXQ6_TIG_STAA8	tig	Trigger factor
Q2FXZ2_DNAK_STAA8	dnaK	Chaperone protein DnaK
Q2FWN4_CH60_STAA8	groEL	60 kDa chaperonin
Q2FZS8_Q2FZS8_STAA8	clpB	ATP-dependent Clp protease, ATP-binding subunit ClpB
Q2G0P5_CLPC_STAA8	clpC	ATP-dependent Clp protease ATP-binding subunit ClpC
Q2FV74_CLPL_STAA8	clpL	ATP-dependent Clp protease ATP-binding subunit ClpL
Q2FXQ7_CLPX_STAA8	clpX	ATP-dependent Clp protease ATP-binding subunit ClpX
Q2G0R0_Q2G0R0_STAA8	ftsH	ATP-dependent zinc metalloprotease FtsH
Q2FXH9_PEPVL_STAA8	SAOUHSC_01868	Dipeptidase PepV
Cell division		
Q2FXK8_EZRA_STAA8	ezrA	Septation ring formation regulator EzrA
O07325_FTSA_STAA8	ftsA	Cell division protein FtsA
Q2FZ89_FTSZ_STAA8	ftsZ	Cell division protein FtsZ
Transcription, translation^*b*^		
Q2G0N5_RPOC_STAA8	rpoC	DNA-directed RNA polymerase subunit beta'
P47768_RPOB_STAA8	rpoB	DNA-directed RNA polymerase subunit beta
Q2FW32_RPOA_STAA8	rpoA	DNA-directed RNA polymerase subunit alpha
Q2G2D0_IF2_STAA8	infB	Translation initiation factor IF-2
Q2G0N1_EFG_STAA8	fusA	Elongation factor G fusA
Q2G0N0_EFTU_STAA8	tuf	Elongation factor Tu
Q2FZ23_EFTS_STAA8	tsf	Elongation factor Ts
Q2G1Y6_Q2G1Y6_STAA8	SAOUHSC_01058	GTP-binding protein TypA, putative

^*a*^ Top hits of proteins identified with unambiguous coverage and found in the SecA_TW-STREP_ but not the untagged SecA sample are shown.

^*b*^ Ribosomal proteins were not listed in the table.

## DISCUSSION

In this study, we identified genetic requirements for secretion of the YSIRK motif-containing protein Geh in *S. aureus* using a screening approach based on isolation of temperature-sensitive mutants with conditional defects in protein secretion. When the *sec* genes were first identified in *E. coli*, *in vitro* experiments examining the translocation of purified precursors into *E. coli* proteoliposomes or using inverted membrane vesicles identified SecA and SecYE as components necessary and sufficient to bind and push precursors (SecA) across the membrane channel (SecYE) (35, 36). Signal peptidase was also found to be essential for cleaving newly translocated precursors and release mature polypeptides (37). Additional factors, while not strictly required, were found to facilitate protein secretion. For example, chaperones, including the secretion-specific chaperone, SecB (38), heat shock proteins (DnaK/DnaJ/GrpE) (39), and trigger factor, a peptidyl-prolyl isomerase (40), maintain preproteins in a secretion competent state (41, 42). SecG in the SecYEG translocon helps increase the rate of translocation (43, 44), while the SecDF/YajC complex assists in stabilizing interactions between SecA-bound precursors and translocon (44, 45).

Gram-positive bacteria harbor *sec* genes reminiscent of those of *E. coli* with some variations. *S. aureus* encodes SecA, SecY, SecE, SecG, SecDF, and YajC, the signal peptidase SpsB, and the additional paralogs SecA2 and SecY2 (46). With the help of accessory secretion proteins, SecA2/SecY2 only transports one substrate, the heavily glycosylated SraP protein (47). Notably, the chaperone protein SecB is missing in *S. aureus* (42, 46). Approximately a third of the *S. aureus* proteome is composed of preproteins with topogenic sequences for secretion across the plasma membrane (48). A subset of these proteins is retained in the envelope by covalent linkage to the peptidoglycan (49–53). These are known as cell wall-anchored (surface) proteins; in addition to an N-terminal signal peptide, surface proteins share a C-terminal LPXTG motif recognized by sortase A. Because surface proteins are immobilized in the envelope, their cellular trafficking can be monitored by microscopy revealing two categories based on signal peptide composition. The presence of a YSIRKxxxGxxS (YSIRK) motif in the signal peptide targets the secretion of 16 surface proteins into the cross-wall during cell division. The remaining five precursors with canonical signal peptides are deposited at the cell poles (25, 54). In *S. aureus*, glycerol ester hydrolase is another secreted protein with a YSIRK motif that is not attached to peptidoglycan (23). Prior studies using genetic depletion of *secA* suggested that SecA is required for the secretion of preproteins with and without a YSIRK motif (55).

Here, a set of temperature-sensitive mutants was isolated to examine genetic requirements for protein secretion in an unbiased manner. Out of 210 temperature-sensitive strains isolated, only a handful displayed defects in secretion of Geh and/or or other Sec pathway substrates. The biggest limitation of our screening approach was the large number of SNPs per isolate revealed by whole-genome sequencing, which were often too numerous to deconvolute and included mutations in DNA repair enzymes that likely exacerbated genetic instability. Nonetheless, this crude mutagenesis identified the temperature-sensitive TS347 isolate and revealed amino acid substitution (G187D) in the ATPase domain of SecA as the basis for the isolate’s concomitant restricted growth and general secretion defect at elevated temperature. Using a western blot analysis, SecA_G187D_ was shown to be unstable at nonpermissive temperatures. Thus, although challenging, this screening approach identified a key genetic component of the secretion pathway.

Two *ts* isolates carried SNPs in *secG*. SecG is found in a complex with SecY and SecE and is conserved in *Escherichia coli*, *Bacillus subtilis*, and *S. aureus* (27–29). In *E. coli*, SecG has been shown to undergo a cycle of topological inversion that mirrors the insertion and de-insertion of SecA across the membrane as it pushes precursors through the SecYEG translocon (56, 57). In *S. aureus*, Sibbald and colleagues reported the complete deletion of *secG* in strain RN4220 and its isogenic *secY2* variant and examined the exoproteomes of these strains using two-dimensional PAGE (27). This analysis revealed the reduced secretion of only eleven proteins in mutants lacking *secG* but profound changes in the exoproteome of a double *secG/secY2* mutant (but not the *secY2* mutant) (27). These observations are in agreement with the notion that SecG facilitates the secretion of some precursors but does not select these substrates based on the YSIRK motif. Here, SNPs identified in the *secG* genes did not contribute to reduced growth at nonpermissive temperature or impaired secretion of Geh.

Isolate TS291 displayed a selective YSIRK secretion defect and bore an amino acid substitution in LtaA previously implicated in regulation of YSIRK protein secretion at the cross-wall (26). However, the isolate’s YSIRK-specific secretion defect could not be attributed to a loss of function of LtaA. It is possible that such functional loss is the result of synthetic interactions between two nonessential proteins. We note that both TS291 and TS92 harbor mutations in the autolysin AtlA and TS205 in the cell wall hydrolase LytN. Both AltA and LytN have been shown to influence the release of proteins from the envelope, albeit that a clear picture reconciling partial peptidoglycan degradation and protein trafficking has yet to emerge (58–62). Undoubtedly, further experiments are needed to deconvolute the complex genetic makeup of TS291 and pinpoint those factors that may specifically influence YSIRK protein secretion.

Isolate TS347 provided an opportunity to assess the activity of SecA_TW-STREP_ via complementation of the *ts* phenotype and implement a pull-down approach for the identification of factors associated with SecA in *S. aureus*. One hypothesis is that SecA may distinguish precursors with the YSIRK motif assisted by a yet unknown factor. The pull-down experiment identified all core ribosomal proteins, trigger factor also referred to as universal holdase, and the cytosolic DnaK and GroEL chaperones. In the well-studied *E. coli* system, secretory preproteins bound by SecA have inherent non-folded properties, but their solubility can further be assisted by general chaperones as well as ribosome-associated trigger factor (42, 63, 64). Furthermore, in *E. coli*, SecA acts as a general chaperone (65) but can also remain associated with ribosome near the polypeptide exit channel to efficiently guide some precursors into the translocation pathway (66). Thus, it appears that a similarly close coordination among ribosomes, chaperones, and SecA may operate in the cytosol of *S. aureus* to maintain Sec pathway substrates in a secretion-competent conformation. Lastly, PepV, a protein annotated as a dipeptidase, was also found to co-purify with SecA_TW-STREP_. This seemed particularly interesting as an SNP was also identified in the corresponding gene in isolate TS30, nominating PepV as a candidate for further studies.

## MATERIALS AND METHODS

### Media and growth conditions

Unless otherwise noted, *S. aureus* strains were grown in tryptic soy broth (TSB) or agar (TSA) with media containing 10 µg/mL chloramphenicol for plasmid selection when needed. *E. coli* was grown on LB medium or agar with 100 µg/mL ampicillin for plasmid selection.

### Bacterial strains and plasmids

Strains and plasmids used in this study are listed in Table S3. Complementation research was conducted using the shuttle vector pSEW016 with the inbuilt Shine-Dalgarno and promoter sequences of the *S. aureus hprK* gene (67). pSEW016-based plasmids, p*secA*, ps*ecA*_G187D_, and p*secG*, were constructed by cloning polymerase chain reaction fragments which for *secA* were amplified with the primer pair 5′-GCGGAGCTCAGGAGGAGCGAACGAAATGGGATT-3′ and 5′-GCGGGATCCTTATTTTCCATGGCAATTTTTGA-3′ using genomic DNA from *S. aureus* RN4220 and TS347, respectively, and for *secG* with primer pair 5′- GCGGCGGAGCTCAGGAGGAGGACAATTTATGCATACATTTTTAATCG-3′ and 5′- GCGGCGGGATCCTTACATACCAAGATAACTTATGC-3′. Plasmid p*secA*_TW-STREP_ was constructed similarly using the primer pair 5′-GCGCTCGAGATGGGATTTTTATCAAAAATTCT-3′ and 5′-GCGGGATCCTTTTCCGTGGCAATTTTTGAATT-3′. The amplified DNA fragment was cloned into another pSEW016-based plasmid containing an inbuilt streptactin sequence for C-terminal tagging (68). RN4220 isolates with *secG*_Q40STOP_ or substitution S29G in the uncharacterized gene SAOUHSC_02008 were obtained by allelic replacement using plasmid pKOR1 as described (69).

### Chemical mutagenesis of *S. aureus* and genomic analysis

*S. aureus* RN4220 (10^10^ CFU) was treated with 0.2 mg/mL of N-methyl-N′-nitro-N-nitrosoguanidine (MNNG) for 30 minutes resulting in approximately 50% killing. After quenching the mutagen with sodium phosphate, the culture was plated at room temperature (~20°C). Isolated colonies were streaked on two plates; one was incubated at room temperature and the other at 42°C. For genomic analysis, chromosomal DNA was extracted from the parent RN4220 used for mutagenesis as well as candidate isolates. Genomic DNA was purified using the Promega Wizard DNA extraction kit using the protocol provided by the manufacturer for Gram-positive bacteria. Genomic DNA samples were submitted to the University of Chicago Genomics Core Facility for whole-genome sequencing. Read mapping to the RN4220 genome (NCBI GenBank reference number GCA_000212435.2) and single nucleotide polymorphism calling relative to wild-type RN4220 were performed using Geneious.

### Protein secretion assays and immunoblot

For screening purposes of TS mutants with various growth defects, as shown in Fig. 1 and 2, cultures were grown in TSB for 20 hours at 30°C. Next, the cultures were centrifuged at 15,800 *× g* for 5 minutes. Supernatants were discarded, and cell pellets reconstituted in 5 mL of TSB at room temperature. This was repeated once to resuspend cells in TSB pre-warmed at 30 or 42°C for incubation at these target temperatures until the cultures reached an optical density at 600 nm of 1.0 or up to 3 hours. For all other experiments, cultures were grown overnight at 30°C and diluted (1:100) the next day in fresh broth, pre-warmed at 30 or 42°C. Cultures were grown until optical density at 600 nm of ~0.5. All immunoblot assays were performed using 1 mL of cultures following a centrifugation step at 15,800 *× g* for 5 minutes to separate proteins sedimenting with the cells (C) from soluble proteins in the supernatants (S). Both C and S samples were precipitated with 10% trichloroacetic acid (TCA). C samples were washed with cold acetone and resuspended in 0.5 M Tris-HCl pH 7.0 to lyse cells at 37°C for 1 hour with 50 µg of lysostaphin. Proteins in these samples were precipitated once more with TCA and processed as described above. Dried TCA-precipitated samples were resuspended in sample buffer for separation over SDS-PAGE and subsequent transfer to a PVDF (polyvinylidene difluoride) membrane. PVDF membranes were blocked and incubated with primary antibody for 1 hour at room temperature or at 4°C overnight. After washing with Tris-buffered saline with 0.1% Tween 20 (TBST), membranes were incubated with secondary antibody conjugated with horseradish peroxidase for 1 hour at room temperature, washed three times with TBST, and developed using the Thermo SuperSignal West Pico Plus kit and exposed to autoradiography films. Data presented in Fig. 1C, 2, 4, and 5 were performed at least four times by at least two investigators; representative images are shown.

### Pull-down experiment

Two-liter cultures of *S. aureus* RN4220 carrying plasmid-encoded p*secA*_TW-STREP_ or p*secA* were grown in TSB and pelleted by centrifugation at 8,000 *× g* for 10 minutes. Cells were resuspended in 50 mL of 100 mM Tris-HCl at pH 8.0 with 150 mM NaCl and 1 mM EDTA (column buffer) and lysed by beating with glass microbeads for 30 minutes. Lysates were cleared by centrifugation at 100,000 *× g* at 4°C for 45 minutes and loaded by gravity flow onto 1  mL of Strep-Tactin Sepharose beads preequilibrated with column buffer. Following low stringency washes with 20-volum column buffer, bound proteins were eluted with desthiobiotin (5  mM). For identification of proteins, samples were submitted to the Harvard University Taplin Mass Spectrometry Facility for LC-MS/MS analysis.

## SUPPLEMENTAL MATERIAL

Supplemental figure and tables jb.00433-24-s0001.docx Fig. S1; Tables S1 to S3.

ASM does not own the copyrights to Supplemental Material that may be linked to, or accessed through, an article. The authors have granted ASM a non-exclusive, world-wide license to publish the Supplemental Material files. Please contact the corresponding author directly for reuse.
