# Supplementary material for: Screening a library of temperature-sensitive mutants to identify secretion factors in Staphylococcus aureus
Source: J Bacteriol. 2025 Jan 16;207(2):e00433-24. doi: 10.1128/jb.00433-24 (PMC11841065; doi:10.1128/jb.00433-24)
Supplement: Supplemental figure and tables — Fig. S1; Tables S1 to S3. [file jb.00433-24-s0001.docx]

**SUPPLEMENTAL MATERIAL**

**Screening a library of temperature-sensitive mutants to identify secretion factors in *Staphylococcus aureus***

Owen Leddy,^1^* Amany M. Ibrahim,* Muhammad S. Azam,* Sadie Solomon^2^, Wenqi Yu,^3^ Olaf Schneewind,† Dominique Missiakas#

Department of Microbiology, Howard Taylor Ricketts Laboratory, The University of Chicago, Lemont, Illinois, USA

*These authors contributed equally to this work.

Current addresses: ^1^Department of Biological Engineering, Massachusetts Institute of Technology, Cambridge, Massachusetts, USA; ^2^Carver College of Medicine, University of Iowa, Iowa City, Iowa, USA; ^3^Department of Molecular Biosciences, Center for Antimicrobial Resistance, University of South Florida, Tampa, Florida, USA

**This file contains:**

- Supplementary Figure 1
- Supplementary Tables 1-3

**
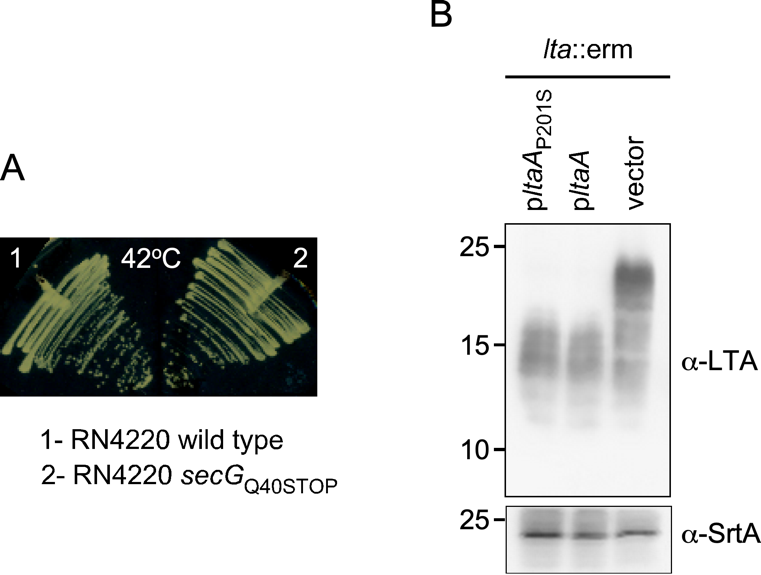
**

**Supplementary Figure 1**: Neither allele *secG*_Q40STOP_ nor *ltaA*_P201S_ are associated with any growth or functional defects. (A) RN4220 wild type or with a *secG*_Q40STOP_ allele were plated at 42°C without loss of viability. (B) The length of LTA was examined by immunoblot following extraction from *ltaA::erm* carrying empty vector, p*ltaA* or p*ltaA*_P201S_ plasmids. Extracts separated on gels prior to transfer and immune detection with antibodies against LTA (αLTA), and SrtA (αSrtA) for loading control.

**Supplementary Table 1:** Sequence analysis of TS isolates (red indicates an essential gene).

| **Isolate number** | **Position** | **Locus Tag** | **Nucleotide Change** | **Amino acid Change** | **Product** | **Protein Effect** |
| --- | --- | --- | --- | --- | --- | --- |
| **TS30** | 1178114 | SAOUHSC_01225 | C to T | S to L | ATP-dependent protease peptidase subunit | Substitution |
|  | 1229282 | SAOUHSC_01272 | C to T | stop codon | DNA mismatch repair protein MutS | Truncation |
|  | 1577242 | SAOUHSC_01663 | G to A | A to V | DNA primase | Substitution |
|  | 1637351 | SAOUHSC_01734 | G to A | G to D | recombination factor protein RarA | Substitution |
|  | 1719745 | SAOUHSC_01813 | C to T | D to N | Predicted transcriptional regulator containing CBS domains | Substitution |
|  | 1730016 | SAOUHSC_01824 | C to T | G to E | thiamine biosynthesis protein ThiI | Substitution |
|  | 1774862 | SAOUHSC_01868 | C to T | G to S | Predicted dipeptidase PepV | Substitution |
|  | 1918175 | SAOUHSC_02008 | C to T | S to N | COG1682, ABC-type polysaccharide/polyol phosphate export permease | Substitution |
|  |  |  |  |  |  |  |
| **TS 92** | 136280 | SAOUHSC_00130 | C to T | E to K | heme-degrading mooxygenase IsdI | Substitution |
|  | 333273 | SAOUHSC_00318 | G to A | A to T | VOC; vicinal oxygen chelate (VOC) family | Substitution |
|  | 467051 | SAOUHSC_00466 | C to T | P to L | isoprenoid synthesis 4-diphosphocytidyl-2C-methyl-D-erythritol kinase | Substitution |
|  | 516747 | SAOUHSC_00514 | G to A | R to Q | Predicted RNA-binding protein containing a PIN domain | Substitution |
|  | 529617 | SAOUHSC_00526 | G to A | E to K | 50S ribosomal protein L7Ae-like protein | Substitution |
|  | 532835 | SAOUHSC_00529 | G to A | E to K | elongation factor G | Substitution |
|  | 965852 | SAOUHSC_00994 | G to A | T to I | bifunctional autolysin AtlA | Substitution |
|  | 1027987 | SAOUHSC_01063 | G to A | A to T | FtsW | Substitution |
|  | 1060813 | SAOUHSC_01099 | G to A | E to K | recombination and DNA strand exchange inhibitor protein | Substitution |
|  | 1187990 | SAOUHSC_01240 | C to T | P to S | prolyl-tRNA synthetase | Substitution |
|  | 1194911 | SAOUHSC_01243 | C to T | P to L | transcription elongation factor NusA | Substitution |
|  | 2116821 | SAOUHSC_02285 | G to A | E to K | 2-isopropylmalate synthase | Substitution |
|  | 2153022 | SAOUHSC_02319 | G to A | V to I | RodA | Substitution |
|  | 2346741 | SAOUHSC_02552 | C to T | G to D | Biotin transporter BioY | Substitution |
|  | 2634743 | SAOUHSC_02862 | C to T | P to S | ATP-dependent Clp protease ATP-binding subunit ClpC | Substitution |
|  |  |  |  |  |  |  |
| **TS 159** | 602528 | SAOUHSC_00611 | C to T | A to V | arginyl-tRNA synthetase | Substitution |
|  | 604406 | SAOUHSC_00612 | C to T | L to F | endonuclease III | Substitution |
|  | 837486 | SAOUHSC_00872 | C to T | A to V | DltD | Substitution |
|  | 1094468 | SAOUHSC_01145 | G to A | V to I | penicillin-binding protein 1 | Substitution |
|  | 1098567 | SAOUHSC_01147 | G to A | D to N | MurD UDP-N-acetylmuramoyl-L-alanyl-D-glutamate synthetase | Substitution |
|  | 1106449 | SAOUHSC_01158 | G to A | R to K | Cell division septum initiation DivIVA, interacts with FtsZ, MinD and other proteins | Substitution |
|  | 1210661 | SAOUHSC_01255 | G to A | E to K | Predicted Zn-dependent peptidase | Substitution |
|  | 1237757 | SAOUHSC_01280 | G to A | S to N | tRNA delta(2)-isopentenylpyrophosphate transferase | Substitution |
|  | 1295913 | SAOUHSC_01352 | G to A | E to K | DNA topoisomerase IV subunit A | Substitution |
|  | 1693879 | SAOUHSC_01797 | G to A | P to S | DNA polymerase I | Substitution |
|  | 1719481 | SAOUHSC_01813 | G to A | stop codon | Predicted transcriptional regulator containing CBS domains | Truncation |
|  | 1997588 | SAOUHSC_02123 | C to T | E to K | ATP-dependent DNA helicase PcrA | Substitution |
|  | 2491087 | SAOUHSC_02709 | C to T | S to F | leukocidin s subunit | Substitution |
|  | 2547035 | SAOUHSC_02770 | G to A | R to C | Diaminopimelate epimerase DapF family | Substitution |
|  | 2553027 | SAOUHSC_02775 | G to A | S to F | Protein of unknown function (DUF1433); | Substitution |
|  |  |  |  |  |  |  |
| **TS 205** | 107554 | SAOUHSC_00103 | C to T | G to D | phosphonates ABC transporter permease | substitution |
|  | 314653 | SAOUHSC_00300 | C to T | stop codon | YSIRK domain-containing triacylglycerol lipase Lip2/Geh (glycerol ester hydrolase ) | truncation |
|  | 784343 | SAOUHSC_00801 | C to T | stop codon | preprotein translocase subunit SecG | truncation |
|  | 1169944 | SAOUHSC_01219 | G to A | D to N | cell wall hydrolase LytN | substitution |
|  | 1657292 | SAOUHSC_01753 | C to T | V to I | GTPase ObgE | substitution |
|  | 2142735 | SAOUHSC_02312 | C to T | stop codon | potassium-transporting ATPase subunit A | truncation |
|  | 2152628 | SAOUHSC_02318 | C to T | C to Y | D-alanyl-alanine synthetase A | substitution |
|  | 2692045 | SAOUHSC_02926 | G to A | E to K | fructose-1,6-bisphosphate aldolase | substitution |
|  | 2700305 | SAOUHSC_02933 | G to A | P to S | betaine aldehyde dehydrogenase | substitution |
|  |  |  |  |  |  |  |
| **TS 291** | 220509 | SAOUHSC_00198 | C to T | G to D | CaiC Acyl-CoA synthetase (AMP-forming)/AMP-acid ligase II | Substitution |
|  | 470694 | SAOUHSC_00471 | C to T | R to C | GlmU family bifunctional N-acetylglucosamine-1-phosphate uridyltransferase | Substitution |
|  | 505006 | SAOUHSC_00504 | C to T | stop codon | ATP:guanido phosphotransferase | Truncation |
|  | 926315 | SAOUHSC_00952 | G to A | P to S | LtaA | Substitution |
|  | 966555 | SAOUHSC_00994 | G to A | P to S | bifunctional autolysin | Substitution |
|  | 1002913 | SAOUHSC_01032 | G to A | G to D | cytochorme d ubiquinol oxidase subunit II | Substitution |
|  | 1011441 | SAOUHSC_01042 | G to A | G to D | branched-chain alpha-keto acid dehydrogenase subunit E2 | Substitution |
|  | 1099788 | SAOUHSC_01148 | G to A | D to N | DivIB cell division protein | Substitution |
|  | 1145600 | SAOUHSC_01193 | G to A | G to D | Predicted kinase related to dihydroxyacetone kinase | Substitution |
|  | 1201065 | SAOUHSC_01249 | G to A | E to K | riboflavin biosynthesis protein RibF | Substitution |
|  | 1222330 | SAOUHSC_01266 | G to A | R to K | 2-oxoacid:acceptor oxidoreductase | Substitution |
|  | 1293697 | SAOUHSC_01351 | G to A | V to I | DNA topoisomerase IV subunit B | Substitution |
|  | 1318730 | SAOUHSC_01374 | G to A | G to D | FemB methicillin resistance factor | Substitution |
|  | 1601280 | SAOUHSC_01690 | C to T | D to N | DNA polymerase III subunit delta | Substitution |
|  | 1641446 | SAOUHSC_01738 | C to T | G to S | histidyl-tRNA syntheatse | Substitution |
|  | 1721944 | SAOUHSC_01815 | C to T | G to E | metal-dependent hydrolase | Substitution |
|  | 1791789 | SAOUHSC_01876 | T to C | T to A | Major Facilitator Superfamily | Substitution |
|  | 1836619 | SAOUHSC_01931 | C to T | A to T | AAA ATPase domain | Substitution |
|  | 1842357 | SAOUHSC_01934 | C to T | A to T | hypothetical protein 44 amino acids | Substitution |
|  | 1877124 | SAOUHSC_01973 | C to T | stop codon | 3'-5' exoribonuclease YhaM | Truncation |
|  | 1881223 | SAOUHSC_01975 | C to T | G to D | DNA repair exonuclease SbcCD nuclease subunit | Substitution |
|  | 2081042 | SAOUHSC_02246 | C to T | G to D | Periplasmic binding protein FeuA | Substitution |
|  | 2110114 | SAOUHSC_02278 | C to T | stop codon | ribosomal-protein-alanine acetyl transferase | Truncation |
|  | 2136529 | SAOUHSC_02305 | C to T | G to D | alanine racemase | Substitution |
|  | 2145706 | SAOUHSC_02314 | C to T | L to F | sensor protein KdpD | Substitution |
|  | 2161214 | SAOUHSC_02330 | C to T | G to D | phosphomethylpyrimidine kinase | Substitution |
|  | 2171297 | SAOUHSC_02345 | C to T | V to I | F0F1 ATP synthase subunit alpha | Substitution |
|  | 2172076 | SAOUHSC_02346 | C to T | G to D | F0F1 ATP synthase subunit delta | Substitution |
|  | 2183248 | SAOUHSC_02362 | C to T | G to D | transcription termiantion factor Rho | Substitution |
|  | 2197567 | SAOUHSC_02374 | C to T | E to K | aminobenzoyl-glutamate utilization protein B | Substitution |
|  | 2216108 | SAOUHSC_02397 | A to G | F to S | ABC transporter ATP-binding protein | Substitution |
|  | 2250847 | SAOUHSC_02423 | C to T | G to D | UDP-N-acetylglucosamine pyrophosphorylase | Substitution |
|  | 2278012 | SAOUHSC_02453 | C to T | V to I | tagatose-6-phosphate kinase | Substitution |
|  | 2311334 | SAOUHSC_02498 | C to T | G to D | 30S ribsomal protein S8 | Substitution |
|  | 2339387 | SAOUHSC_02542 | C to T | D to N | molybdopterin biosynthesis protein MoeA | Substitution |
|  | 2351699 | SAOUHSC_02557 | C to T | V to I | urea transporter | Substitution |
|  | 2397929 | SAOUHSC_02607 | C to T | G to D | urocanate hydratase | Substitution |
|  | 2432457 | SAOUHSC_02647 | C to T | G to E | malate:quinone oxidoreductase | Substitution |
|  | 2521365 | SAOUHSC_02741 | C to T | G to D | amino acid ABC transporter permease | Substitution |
|  | 2616488 | SAOUHSC_02841 | C to T | D to N | Phosphotransferase system, fructose-specific IIC component | Substitution |
|  | 2630420 | SAOUHSC_02859 | C to T | G to S | hydroxymethylglutaryl-CoA reductase | Substitution |
|  | 2643314 | SAOUHSC_02869 | C to T | G to S | 1-pyrroline-5-carboxylate dehydrogenase | Substitution |
|  | 2684189 | SAOUHSC_02919 | C to T | E to K | 3-methyl-2-oxobutanoate hydroxymethyltransferase | Substitution |
|  | 2690506 | SAOUHSC_02924 | C to T | G to D | 4-aminobutyrate aminotransferase | Substitution |
|  | 2705892 | SAOUHSC_02941 | C to T | G to E | 4Fe-4S single cluster domain | Substitution |
|  |  |  |  |  |  |  |
| **TS 347** | 19,240 | SAOUHSC_00015 | G to A | G to D | GdpP; c-di-AMP phosphodiesterase | Substitution |
|  | 65,407 | SAOUHSC_00060 | C to T | P to S | NptA; Na+/phosphate symporter | Substitution |
|  | 391,101 | SAOUHSC_00386 | G to A | E to K | superantigen like | Substitution |
|  | 750,499 | SAOUHSC_00769 | G to A | G to D | preprotein translocase subunit SecA | Substitution |
|  | 784,311 | SAOUHSC_00801 | G to A | G to E | preprotein translocase subunit SecG | Substitution |
|  | 2,474,133 | SAOUHSC_02687 | C to T | S to F | formate/nitrite transporter, putative | Substitution |
|  | 2,585,572 | SAOUHSC_02806 | G to A | G to R | gluconate permease, putative | Substitution |
|  | 2,817,241 | SAOUHSC_03052 | G to A | D to N | GidA; glucose-inhibited cell division protein A | Substitution |
|  | 2,819,878 | SAOUHSC_03053 | G to A | V to M | trmE tRNA modification GTPase | Substitution |

**Supplementary Table 2:** All proteins identified in the SecA_TW-STREP_ but not the untagged SecA sample.

| **Reference** | **Gene Symbol** | **Annotation** | **MW (kDa)** |
| --- | --- | --- | --- |
| O06446_SECA1_STAA8 | secA1 | Protein translocase subunit SecA 1 OS=Staphylococcus aureus (strain NCTC 8325) GN=secA1 PE=1 SV=2 | 95.9 |
| Q2G1D8_PFLB_STAA8 | pflB | Formate acetyltransferase OS=Staphylococcus aureus (strain NCTC 8325) GN=pflB PE=3 SV=1 | 84.81 |
| Q2G2C1_Q2G2C1_STAA8 | SAOUHSC_01064 | Pyruvate carboxylase OS=Staphylococcus aureus (strain NCTC 8325) GN=SAOUHSC_01064 PE=3 SV=1 | 128.47 |
| Q2G0N5_RPOC_STAA8 | rpoC | DNA-directed RNA polymerase subunit beta' OS=Staphylococcus aureus (strain NCTC 8325) GN=rpoC PE=3 SV=2 | 135.32 |
| P47768_RPOB_STAA8 | rpoB | DNA-directed RNA polymerase subunit beta OS=Staphylococcus aureus (strain NCTC 8325) GN=rpoB PE=3 SV=2 | 133.14 |
| Q2G0N1_EFG_STAA8 | fusA | Elongation factor G OS=Staphylococcus aureus (strain NCTC 8325) GN=fusA PE=3 SV=3 | 76.56 |
| Q2G1K9_Q2G1K9_STAA8 | SAOUHSC_00113 | Alcohol dehydrogenase, iron-containing, putative OS=Staphylococcus aureus (strain NCTC 8325) GN=SAOUHSC_00113 PE=4 SV=1 | 94.88 |
| Q2FZ82_SYI_STAA8 | ileS | Isoleucine--tRNA ligase OS=Staphylococcus aureus (strain NCTC 8325) GN=ileS PE=3 SV=1 | 104.82 |
| Q2FXZ2_DNAK_STAA8 | dnaK | Chaperone protein DnaK OS=Staphylococcus aureus (strain NCTC 8325) GN=dnaK PE=3 SV=1 | 66.32 |
| Q2G0N0_EFTU_STAA8 | tuf | Elongation factor Tu OS=Staphylococcus aureus (strain NCTC 8325) GN=tuf PE=3 SV=1 | 43.08 |
| Q2FXK8_EZRA_STAA8 | ezrA | Septation ring formation regulator EzrA OS=Staphylococcus aureus (strain NCTC 8325) GN=ezrA PE=3 SV=1 | 66.21 |
| Q2G2S8_Q2G2S8_STAA8 | SAOUHSC_01974 | Putative uncharacterized protein OS=Staphylococcus aureus (strain NCTC 8325) GN=SAOUHSC_01974 PE=4 SV=1 | 114.35 |
| Q2G0P5_CLPC_STAA8 | clpC | ATP-dependent Clp protease ATP-binding subunit ClpC OS=Staphylococcus aureus (strain NCTC 8325) GN=clpC PE=1 SV=1 | 90.98 |
| Q2G193_Q2G193_STAA8 | SAOUHSC_00253 | Putative uncharacterized protein OS=Staphylococcus aureus (strain NCTC 8325) GN=SAOUHSC_00253 PE=4 SV=1 | 57.89 |
| Q2FZS8_Q2FZS8_STAA8 | SAOUHSC_00912 | ATP-dependent Clp protease, ATP-binding subunit ClpB OS=Staphylococcus aureus (strain NCTC 8325) GN=SAOUHSC_00912 PE=3 SV=1 | 98.27 |
| Q2FWZ0_GATB_STAA8 | gatB | Aspartyl/glutamyl-tRNA(Asn/Gln) amidotransferase subunit B OS=Staphylococcus aureus (strain NCTC 8325) GN=gatB PE=3 SV=1 | 53.62 |
| Q2FVL2_Q2FVL2_STAA8 | SAOUHSC_02699 | Putative uncharacterized protein OS=Staphylococcus aureus (strain NCTC 8325) GN=SAOUHSC_02699 PE=4 SV=1 | 28.89 |
| P95689_SYS_STAA8 | serS | Serine--tRNA ligase OS=Staphylococcus aureus (strain NCTC 8325) GN=serS PE=3 SV=1 | 48.61 |
| Q2FWN4_CH60_STAA8 | groL | 60 kDa chaperonin OS=Staphylococcus aureus (strain NCTC 8325) GN=groL PE=3 SV=1 | 57.63 |
| Q2FV74_CLPL_STAA8 | clpL | ATP-dependent Clp protease ATP-binding subunit ClpL OS=Staphylococcus aureus (strain NCTC 8325) GN=clpL PE=2 SV=1 | 77.79 |
| Q2G2D0_IF2_STAA8 | infB | Translation initiation factor IF-2 OS=Staphylococcus aureus (strain NCTC 8325) GN=infB PE=3 SV=1 | 77.82 |
| Q2FXP7_SYT_STAA8 | thrS | Threonine--tRNA ligase OS=Staphylococcus aureus (strain NCTC 8325) GN=thrS PE=3 SV=1 | 74.44 |
| Q2G188_Q2G188_STAA8 | SAOUHSC_00258 | Putative uncharacterized protein OS=Staphylococcus aureus (strain NCTC 8325) GN=SAOUHSC_00258 PE=4 SV=1 | 114.75 |
| Q2G0R0_Q2G0R0_STAA8 | ftsH | ATP-dependent zinc metalloprotease FtsH OS=Staphylococcus aureus (strain NCTC 8325) GN=ftsH PE=3 SV=1 | 77.76 |
| Q2FZD8_Q2FZD8_STAA8 | pheT | Phenylalanine--tRNA ligase beta subunit OS=Staphylococcus aureus (strain NCTC 8325) GN=pheT PE=3 SV=1 | 88.87 |
| Q2FXH9_PEPVL_STAA8 | SAOUHSC_01868 | Putative dipeptidase SAOUHSC_01868 OS=Staphylococcus aureus (strain NCTC 8325) GN=SAOUHSC_01868 PE=3 SV=1 | 52.79 |
| Q2FXE8_Q2FXE8_STAA8 | SAOUHSC_01901 | Putative uncharacterized protein OS=Staphylococcus aureus (strain NCTC 8325) GN=SAOUHSC_01901 PE=3 SV=1 | 25.69 |
| Q2FW32_RPOA_STAA8 | rpoA | DNA-directed RNA polymerase subunit alpha OS=Staphylococcus aureus (strain NCTC 8325) GN=rpoA PE=3 SV=1 | 34.99 |
| Q2FY60_Q2FY60_STAA8 | SAOUHSC_01605 | 6-phosphogluconate dehydrogenase, decarboxylating OS=Staphylococcus aureus (strain NCTC 8325) GN=SAOUHSC_01605 PE=3 SV=1 | 51.77 |
| Q2FXU5_SYD_STAA8 | aspS | Aspartate--tRNA ligase OS=Staphylococcus aureus (strain NCTC 8325) GN=aspS PE=3 SV=1 | 66.59 |
| Q2FZ23_EFTS_STAA8 | tsf | Elongation factor Ts OS=Staphylococcus aureus (strain NCTC 8325) GN=tsf PE=3 SV=1 | 32.47 |
| Q2G1Y6_Q2G1Y6_STAA8 | SAOUHSC_01058 | GTP-binding protein TypA, putative OS=Staphylococcus aureus (strain NCTC 8325) GN=SAOUHSC_01058 PE=4 SV=1 | 69.15 |
| Q2G264_Q2G264_STAA8 | SAOUHSC_01180 | Putative uncharacterized protein OS=Staphylococcus aureus (strain NCTC 8325) GN=SAOUHSC_01180 PE=4 SV=1 | 35.88 |
| Q2FW18_RL5_STAA8 | rplE | 50S ribosomal protein L5 OS=Staphylococcus aureus (strain NCTC 8325) GN=rplE PE=3 SV=1 | 20.25 |
| Q2FZ89_FTSZ_STAA8 | ftsZ | Cell division protein FtsZ OS=Staphylococcus aureus (strain NCTC 8325) GN=ftsZ PE=3 SV=1 | 41.01 |
| Q2FXZ9_Y1676_STAA8 | SAOUHSC_01676 | UPF0365 protein SAOUHSC_01676 OS=Staphylococcus aureus (strain NCTC 8325) GN=SAOUHSC_01676 PE=3 SV=1 | 35.16 |
| Q2G2H4_Q2G2H4_STAA8 | SAOUHSC_00002 | DNA polymerase III subunit beta OS=Staphylococcus aureus (strain NCTC 8325) GN=SAOUHSC_00002 PE=3 SV=1 | 41.89 |
| Q2FV17_ALF1_STAA8 | fda | Fructose-bisphosphate aldolase class 1 OS=Staphylococcus aureus (strain NCTC 8325) GN=fda PE=3 SV=1 | 33.03 |
| Q2FVK5_SBI_STAA8 | sbi | Immunoglobulin-binding protein sbi OS=Staphylococcus aureus (strain NCTC 8325) GN=sbi PE=1 SV=1 | 50.04 |
| Q2FWC1_PDP_STAA8 | pdp | Pyrimidine-nucleoside phosphorylase OS=Staphylococcus aureus (strain NCTC 8325) GN=pdp PE=3 SV=1 | 46.28 |
| Q2FZK7_ATL_STAA8 | atl | Bifunctional autolysin OS=Staphylococcus aureus (strain NCTC 8325) GN=atl PE=1 SV=1 | 137.3 |
| Q2G1Z4_SYP_STAA8 | proS | Proline--tRNA ligase OS=Staphylococcus aureus (strain NCTC 8325) GN=proS PE=3 SV=1 | 63.82 |
| Q2FYM1_ODO1_STAA8 | odhA | 2-oxoglutarate dehydrogenase E1 component OS=Staphylococcus aureus (strain NCTC 8325) GN=odhA PE=3 SV=1 | 105.28 |
| Q2G1B7_Q2G1B7_STAA8 | SAOUHSC_00228 | Putative uncharacterized protein OS=Staphylococcus aureus (strain NCTC 8325) GN=SAOUHSC_00228 PE=4 SV=1 | 66.27 |
| Q2FWY9_GATA_STAA8 | gatA | Glutamyl-tRNA(Gln) amidotransferase subunit A OS=Staphylococcus aureus (strain NCTC 8325) GN=gatA PE=3 SV=1 | 52.79 |
| Q2G2D7_Q2G2D7_STAA8 | SAOUHSC_02447 | Putative uncharacterized protein OS=Staphylococcus aureus (strain NCTC 8325) GN=SAOUHSC_02447 PE=4 SV=1 | 36.24 |
| Q2FZH5_Q2FZH5_STAA8 | SAOUHSC_01029 | Phosphoenolpyruvate-protein phosphotransferase OS=Staphylococcus aureus (strain NCTC 8325) GN=SAOUHSC_01029 PE=3 SV=1 | 63.18 |
| Q2FXV9_SYA_STAA8 | alaS | Alanine--tRNA ligase OS=Staphylococcus aureus (strain NCTC 8325) GN=alaS PE=3 SV=1 | 98.46 |
| Q2FYF9_Q2FYF9_STAA8 | SAOUHSC_01493 | 30S ribosomal protein S1, putative OS=Staphylococcus aureus (strain NCTC 8325) GN=SAOUHSC_01493 PE=4 SV=1 | 43.26 |
| Q2FYT8_Q2FYT8_STAA8 | SAOUHSC_01337 | Transketolase OS=Staphylococcus aureus (strain NCTC 8325) GN=SAOUHSC_01337 PE=4 SV=1 | 68.32 |
| Q2FY08_SYG_STAA8 | glyQS | Glycine--tRNA ligase OS=Staphylococcus aureus (strain NCTC 8325) GN=glyQS PE=3 SV=1 | 53.59 |
| Q2G0F8_SYR_STAA8 | argS | Arginine--tRNA ligase OS=Staphylococcus aureus (strain NCTC 8325) GN=argS PE=3 SV=1 | 62.34 |
| Q2FYQ2_Q2FYQ2_STAA8 | SAOUHSC_01383 | Putative uncharacterized protein OS=Staphylococcus aureus (strain NCTC 8325) GN=SAOUHSC_01383 PE=4 SV=1 | 69.24 |
| Q2FXN9_Q2FXN9_STAA8 | SAOUHSC_01797 | DNA polymerase OS=Staphylococcus aureus (strain NCTC 8325) GN=SAOUHSC_01797 PE=3 SV=1 | 99.13 |
| Q2FZ25_RS2_STAA8 | rpsB | 30S ribosomal protein S2 OS=Staphylococcus aureus (strain NCTC 8325) GN=rpsB PE=3 SV=2 | 29.08 |
| Q2FZT4_Y906_STAA8 | SAOUHSC_00906 | Uncharacterized protein SAOUHSC_00906 OS=Staphylococcus aureus (strain NCTC 8325) GN=SAOUHSC_00906 PE=3 SV=1 | 33.09 |
| Q2FWH3_DDL_STAA8 | ddl | D-alanine--D-alanine ligase OS=Staphylococcus aureus (strain NCTC 8325) GN=ddl PE=3 SV=1 | 40.21 |
| Q2G245_Q2G245_STAA8 | SAOUHSC_01854 | Putative uncharacterized protein OS=Staphylococcus aureus (strain NCTC 8325) GN=SAOUHSC_01854 PE=4 SV=1 | 55.06 |
| Q2FXR8_SYV_STAA8 | valS | Valine--tRNA ligase OS=Staphylococcus aureus (strain NCTC 8325) GN=valS PE=3 SV=1 | 101.66 |
| Q2FWE8_ATPA_STAA8 | atpA | ATP synthase subunit alpha OS=Staphylococcus aureus (strain NCTC 8325) GN=atpA PE=3 SV=1 | 54.55 |
| Q2FXQ6_TIG_STAA8 | tig | Trigger factor OS=Staphylococcus aureus (strain NCTC 8325) GN=tig PE=3 SV=1 | 48.58 |
| Q2FWP0_LUKL1_STAA8 | SAOUHSC_02241 | Uncharacterized leukocidin-like protein 1 OS=Staphylococcus aureus (strain NCTC 8325) GN=SAOUHSC_02241 PE=1 SV=1 | 38.66 |
| Q2FWN9_LUKL2_STAA8 | SAOUHSC_02243 | Uncharacterized leukocidin-like protein 2 OS=Staphylococcus aureus (strain NCTC 8325) GN=SAOUHSC_02243 PE=3 SV=1 | 40.41 |
| Q2FWF4_Q2FWF4_STAA8 | murA | UDP-N-acetylglucosamine 1-carboxyvinyltransferase OS=Staphylococcus aureus (strain NCTC 8325) GN=murA PE=3 SV=1 | 44.91 |
| Q2FXT8_Q2FXT8_STAA8 | SAOUHSC_01746 | Protein-export membrane protein SecDF OS=Staphylococcus aureus (strain NCTC 8325) GN=SAOUHSC_01746 PE=3 SV=1 | 82 |
| Q2FXL1_THII_STAA8 | thiI | Probable tRNA sulfurtransferase OS=Staphylococcus aureus (strain NCTC 8325) GN=thiI PE=3 SV=1 | 46.18 |
| Q2FXQ7_CLPX_STAA8 | clpX | ATP-dependent Clp protease ATP-binding subunit ClpX OS=Staphylococcus aureus (strain NCTC 8325) GN=clpX PE=3 SV=1 | 46.27 |
| Q2FWD4_Q2FWD4_STAA8 | murA | UDP-N-acetylglucosamine 1-carboxyvinyltransferase OS=Staphylococcus aureus (strain NCTC 8325) GN=murA PE=3 SV=1 | 45.05 |
| Q2G2S6_PRSA_STAA8 | prsA | Foldase protein PrsA OS=Staphylococcus aureus (strain NCTC 8325) GN=prsA PE=3 SV=1 | 35.62 |
| Q2G1B8_Q2G1B8_STAA8 | SAOUHSC_00227 | Putative uncharacterized protein OS=Staphylococcus aureus (strain NCTC 8325) GN=SAOUHSC_00227 PE=4 SV=1 | 66.03 |
| Q2FYL3_Q2FYL3_STAA8 | SAOUHSC_01427 | Putative uncharacterized protein OS=Staphylococcus aureus (strain NCTC 8325) GN=SAOUHSC_01427 PE=3 SV=1 | 55.23 |
| Q2FZ58_Y1193_STAA8 | SAOUHSC_01193 | Uncharacterized protein SAOUHSC_01193 OS=Staphylococcus aureus (strain NCTC 8325) GN=SAOUHSC_01193 PE=4 SV=1 | 60.48 |
| Q2FZ92_MURD_STAA8 | murD | UDP-N-acetylmuramoylalanine--D-glutamate ligase OS=Staphylococcus aureus (strain NCTC 8325) GN=murD PE=3 SV=1 | 49.81 |
| Q2FW12_RS3_STAA8 | rpsC | 30S ribosomal protein S3 OS=Staphylococcus aureus (strain NCTC 8325) GN=rpsC PE=3 SV=1 | 24.09 |
| Q2FXJ6_Q2FXJ6_STAA8 | SAOUHSC_01838 | Putative uncharacterized protein OS=Staphylococcus aureus (strain NCTC 8325) GN=SAOUHSC_01838 PE=4 SV=1 | 45.78 |
| Q2G1B9_Q2G1B9_STAA8 | SAOUHSC_00226 | Putative uncharacterized protein OS=Staphylococcus aureus (strain NCTC 8325) GN=SAOUHSC_00226 PE=4 SV=1 | 38.43 |
| Q2G0Q3_SYK_STAA8 | lysS | Lysine--tRNA ligase OS=Staphylococcus aureus (strain NCTC 8325) GN=lysS PE=3 SV=1 | 56.68 |
| Q2G1R9_Q2G1R9_STAA8 | metG | Methionine--tRNA ligase OS=Staphylococcus aureus (strain NCTC 8325) GN=metG PE=3 SV=1 | 74.84 |
| Q2FW23_RS5_STAA8 | rpsE | 30S ribosomal protein S5 OS=Staphylococcus aureus (strain NCTC 8325) GN=rpsE PE=3 SV=1 | 17.73 |
| Q2G115_Q2G115_STAA8 | ychF | Ribosome-binding ATPase YchF OS=Staphylococcus aureus (strain NCTC 8325) GN=ychF PE=3 SV=1 | 40.57 |
| O07325_FTSA_STAA8 | ftsA | Cell division protein FtsA OS=Staphylococcus aureus (strain NCTC 8325) GN=ftsA PE=1 SV=2 | 52.9 |
| Q2FZP6_MURE_STAA8 | murE | UDP-N-acetylmuramoyl-L-alanyl-D-glutamate--L-lysine ligase OS=Staphylococcus aureus (strain NCTC 8325) GN=murE PE=1 SV=1 | 54.07 |
| Q2FZ31_TRMFO_STAA8 | trmFO | Methylenetetrahydrofolate--tRNA-(uracil-5-)-methyltransferase TrmFO OS=Staphylococcus aureus (strain NCTC 8325) GN=trmFO PE=3 SV=1 | 48.34 |
| Q2FWH5_Y2316_STAA8 | SAOUHSC_02316 | Probable DEAD-box ATP-dependent RNA helicase SAOUHSC_02316 OS=Staphylococcus aureus (strain NCTC 8325) GN=SAOUHSC_02316 PE=3 SV=1 | 56.91 |
| Q2G0P0_RL1_STAA8 | rplA | 50S ribosomal protein L1 OS=Staphylococcus aureus (strain NCTC 8325) GN=rplA PE=3 SV=1 | 24.69 |
| Q2G2D2_Q2G2D2_STAA8 | nusA | Transcription termination/antitermination protein NusA OS=Staphylococcus aureus (strain NCTC 8325) GN=nusA PE=3 SV=1 | 43.71 |
| Q2FYH6_SYN_STAA8 | asnS | Asparagine--tRNA ligase OS=Staphylococcus aureus (strain NCTC 8325) GN=asnS PE=3 SV=1 | 49.13 |
| Q2FYL5_MURG_STAA8 | murG | UDP-N-acetylglucosamine--N-acetylmuramyl-(pentapeptide) pyrophosphoryl-undecaprenol N-acetylglucosamine transferase OS=Staphylococcus aureus (strain NCTC 8325) GN=murG PE=3 SV=1 | 39.67 |
| Q2FXI5_Q2FXI5_STAA8 | SAOUHSC_01861 | Putative uncharacterized protein OS=Staphylococcus aureus (strain NCTC 8325) GN=SAOUHSC_01861 PE=4 SV=1 | 39.76 |
| Q2FW16_RL14_STAA8 | rplN | 50S ribosomal protein L14 OS=Staphylococcus aureus (strain NCTC 8325) GN=rplN PE=3 SV=1 | 13.13 |
| P60430_RL2_STAA8 | rplB | 50S ribosomal protein L2 OS=Staphylococcus aureus (strain NCTC 8325) GN=rplB PE=3 SV=1 | 30.14 |
| Q2FZW3_Q2FZW3_STAA8 | SAOUHSC_00872 | Extramembranal protein OS=Staphylococcus aureus (strain NCTC 8325) GN=SAOUHSC_00872 PE=4 SV=1 | 44.92 |
| Q2FW21_RL6_STAA8 | rplF | 50S ribosomal protein L6 OS=Staphylococcus aureus (strain NCTC 8325) GN=rplF PE=3 SV=1 | 19.77 |

**Supplementary Table 3:** Plasmids and strains used in this study.

| **Strain/plasmids** | **Description** | **Source** |
| --- | --- | --- |
| **Vectors and plasmids** | | |
| pSEW016 | *E. coli*/*S. aureus* shuttle vector | Laboratory collection |
| pKOR1 | Allelic replacement vector | Laboratory |
| p*SecA* | *secA* gene cloned into pSEW016 | This study |
| p*SecA*_G187D_ | *secA_G187D_* allele cloned into pSEW016 | This study |
| p*SecA*_TW-STREP_ | *SecA*_TW-STREP_ allele cloned into pSEW016 | This study |
| p*SecG* | *secG* gene cloned into pSEW016 | This study |
| p*secG*_Q40STOP_ | *secG*_Q40STOP_ substitution cloned into pKOR1 | This study |
| **Strains** | | |
| DH5α | *E. coli* cloning strain | Laboratory collection |
| RN4220 (WT, wild type) | *S. aureus* laboratory strain | Laboratory collection |
| Δ*geh* | RN4220 lacking *geh* | Laboratory collection |
| Δ*nuc* | RN4220 lacking *nuc* | Laboratory collection |
| TS30 | Mutagenized RN4220. Sup. Table 1 for description | Isolate was lost during the course of the study |
| TS92 | Mutagenized RN4220. Sup. Table 1 for description | This study |
| TS159 | Mutagenized RN4220. Sup. Table 1 for description | This study |
| TS205 | Mutagenized RN4220. Sup. Table 1 for description | This study |
| TS291 | Mutagenized RN4220. Sup. Table 1 for description | This study |
| TS347 | Mutagenized RN4220. Sup. Table 1 for description | This study |
